# Generation of evidence-based carboplatin dosing guidelines for neonates and infants

**DOI:** 10.1038/s41416-023-02456-y

**Published:** 2023-10-10

**Authors:** Shelby Barnett, Guy Makin, Deborah A. Tweddle, Caroline Osborne, Gareth J. Veal

**Affiliations:** 1 Translational & Clinical Research Institute, Newcastle University Centre for Cancer, Newcastle University, Newcastle upon Tyne, UK; 2 Division of Cancer Sciences, University of Manchester, Manchester, UK; 3 Royal Manchester Children’s Hospital, Manchester, UK; 4 Great North Children’s Hospital, Newcastle upon Tyne, UK; 5 Pharmacy Department, Alder Hey Children’s NHS Foundation Trust, Liverpool, UK

**Keywords:** Chemotherapy, Paediatric research

## Abstract

**Background:**

To optimally dose childhood cancer patients it is essential that we apply evidence-based dosing approaches. Carboplatin is commonly dosed to achieve a cumulative target exposure (AUC) in children, with target AUC values of 5.2–7.8 mg/ml.min defined. To achieve these exposures patients are dosed at 6.6 mg/kg/day or 4.4 mg/kg for patients <5 kg. The current study uses real world clinical pharmacology data to optimise body weight-based doses to effectively target AUCs of 5.2–7.8 mg/ml.min in infants.

**Methods:**

Carboplatin exposures were determined across 165 treatment cycles in 82 patients ≤10 kg. AUC and clearance values were determined by Bayesian modelling from samples collected on day 1. These parameters were utilised to assess current dosing variability, determine doses required to achieve target AUC values and predict change in AUC using the modified dose.

**Results:**

No significant differences in clearance were identified between patients <5 kg and 5–10 kg. Consequently, for patients <5 kg, 4.4 mg/kg dosing was not sufficient to achieve a target AUC of 5.2 mg/ml.min, with <55% of patients within 25% of this target. Optimised daily doses for patients ≤10 kg were 6 mg/kg and 9 mg/kg for cumulative carboplatin target exposures of 5.2 and 7.8 mg/ml.min, respectively.

**Conclusions:**

Adoption of these evidence-based carboplatin doses in neonates and infants will reduce drug exposure variability and positively impact treatment.

## Background

Infant and neonate childhood cancer patients represent a challenging patient population for a variety of reasons. The dosing of anticancer drugs in these patients is historically carried out using body weight-based dosing but there is limited scientific rationale for this approach. The dearth of information in this area and the importance of generating evidence-based dosing guidance has recently been highlighted [1–3].

Carboplatin is a widely used antineoplastic agent in childhood cancer, but its use is associated with a range of potentially life changing toxicities including nephrotoxicity, ototoxicity and peripheral neuropathy [4–6]. There is a well-understood relationship between carboplatin exposure as defined by area under the concentration time curve (AUC) and both efficacy and toxicity. Carboplatin exposures above 8 mg/ml.min have no additional impact on efficacy in adults [7], with exposures of >6.5–7 mg/ml.min associated with increased incidence of thrombocytopenia in children [8, 9]. Therefore, for standard dose carboplatin therapy a drug exposure of 7.8 mg/ml.min is commonly targeted in both adult and paediatric patients, to maximise the chance of efficacy with acceptable toxicity. However, a reduced AUC of 5.2 mg/ml.min is usually targeted for infant patients, largely due to concerns over the potential for harmful side-effects of treatment in this susceptible patient population and use of other chemotherapies alongside carboplatin. For example the incidence of ototoxicity has been shown to be increased in patients <6 months [4]. If this lower exposure is well tolerated in infants the target can be increased to 7.8 mg/ml.min, with a view to improving clinical outcome. To date this evidence has largely come from experiences with relatively small numbers of patients being studied in a therapeutic drug monitoring (TDM) setting in the United Kingdom [10].

Carboplatin doses in adults are routinely calculated based on measured GFR values according to well established formulae [11]. Although paediatric GFR-based dosing formulae exist, standard practice for dosing carboplatin in paediatric patients in the UK involves body weight or surface area dosing approaches [12]. This is partly a result of the challenges associated with obtaining reliable GFR estimates in neonate and infant patients. BSA based dosing has previously been associated with excessive carboplatin toxicity in very young patients [4]. In addition, the benefits of weight-based dosing of carboplatin have recently been shown in a study involving the re-evaluation of childhood dosing formulae in children 1–8 years of age [13]. As is the case for many anticancer drugs, dose reductions in the very young are implemented if a patient is under a defined age or weight, but these modifications are largely based on arbitrary cut-off values as opposed to sound pharmacological evidence. A summary of commonly used protocol-based dosing regimens and dose reductions for infants is shown in Table 1, with a more detailed analysis provided in a recent carboplatin review [12]. Typically when dosing infant patients, doses of 6.6 mg/kg/day over 3 days are utilised for patients <10 kg, with a 33% dose reduction to 4.4 mg/kg/day applied to patients <5 kg. Alternatively, a percent reduction from 200 mg/m^2^/day dosing may be used in some protocols (Table 1).

**Table 1:** Examples of carboplatin dose adjustments utilised in infant cancer treatment protocols in the UK.

Tumour type	Standard dose	Dose adjustment	Target AUC (mg/ml.min)	Ref
Renal tumours (Umbrella study protocol)	200 mg/m^2^ (for 3 days)	For children 5–12 kg: Reduce to 6.6 mg/kg For children <5 kg: Reduce to 4.4 mg/kg	5.2–7.95	[22]
Low/intermediate-risk neuroblastoma	200 mg/m^2^ (for 3 days)	For children 5–10 kg: Reduce to 6.6 mg/kg For children <5 kg: Reduce to 4.4 mg/kg	5.2–7.8	[16]
Retinoblastoma	200 mg/m^2^ (for 3 days)	Start at 4.4 mg/kg and if well tolerated increase to 6.6 mg/kg	5.2–7.8	[15]
Infant medulloblastoma	200 mg/m^2^ (for 3 days)	0–5 months 66% carboplatin BSA dose 6–11 months 80% carboplatin BSA dose 12+ months full dose	7.8	[23]
Choroid plexus tumours	235 mg/m^2^ (for 3 days)	<1 year 6.6 mg/kg <3 months 4.4 mg/kg	7.8 (<1 year) 5.2 (<3 months)	[24]

In the United Kingdom, carboplatin real-time dose intervention is supported by a national TDM programme of work [12, 14]. The Children’s Cancer and Leukaemia Group (CCLG) recommends TDM as standard of care in infants for several tumour types, including choroid plexus tumours, neuroblastoma and retinoblastoma [15, 16]. Currently, approximately 50 paediatric patients benefit from carboplatin TDM each year; these include neonates, infants and high dose chemotherapy patients. Multiple cycles of carboplatin therapy are often monitored for infant patients, as renal function matures significantly within the first weeks and months of life. Therefore, carboplatin doses can change markedly between cycles in a TDM setting, in order for patients to achieve target AUC values on consecutive cycles of treatment [10].

The current study aims to assess current carboplatin dosing practices in infant cancer patients and use real world data generated from carboplatin TDM studies to provide evidence-based dosing regimens designed to achieve desired carboplatin exposures in this challenging patient population.

## Methods

### Patients and treatment

Real-time carboplatin data from patients ≤10 kg, targeting cumulative AUC values of 5.2 and 7.8 mg/ml.min over three days of treatment, were included in the analysis. A total 82 patients met these criteria; informed consent was given and patients were recruited as part of the NCCPG TDM 2018 study (ISRCTN 10139334; REC 18/NE/0384) or were historical in house data from clinical requests. This patient cohort contained both pre-term and full term infants, age in this analysis refers to postnatal age. On days 1 and 2 of treatment, patients received carboplatin doses of either 4.4 mg/kg/day (<5 kg) or 6.6 mg/kg/day (5–10 kg), based on infant treatment protocol guidelines, to achieve a cumulative AUC of 5.2 mg/ml.min. For patients targeting a higher AUC of 7.8 mg/ml.min, an initial daily dose of 6.6 mg/kg was used. A small number of patients were dosed using a percent reduction from BSA-based dosing (200 mg/m^2^), but when normalised to body weight these doses were equivalent to approximately 6.6 mg/kg. Carboplatin doses on day 3 were adjusted accordingly to ensure attainment of the defined cumulative carboplatin AUC.

### Sample analysis

Blood samples were taken on day 1 from patients at the following time points: mid-infusion, end of carboplatin infusion and 1–2 h post end of infusion. Blood samples were immediately spun to plasma in a pre-cooled (4 °C) centrifuge at 1200 × *g* for 5 min. Approximately 0.5 mL of plasma was then removed and transferred to an Amicon Centrifree micropartition unit with a 30,000 MW cut-off (Millipore, Edinburgh, UK) and spun at 1500 g pre-cooled (4 °C) centrifuge for 15 minutes. This process was conducted to obtain the free fraction of carboplatin in the plasma ultrafiltrate (UF), which were immediately frozen and shipped overnight on dry ice for analysis on day 2 of patient treatment. Patients received the same dose of carboplatin on day 2, with a view to making required dose adjustments on day 3 to achieve the target cumulative AUC. Carboplatin UF concentrations were determined using flameless atomic absorption spectrophotometry (AAS) as described previously [17].

### Pharmacokinetic analysis

Bayesian analysis was used to determine carboplatin AUC and clearance (CL), using a two-compartment model as previously described [18]. Based on previously obtained data it was assumed that carboplatin exposures on day 1 and 2 would be the same, following administration of the same daily dose. Therefore using the target cumulative exposure, and the AUC achieved on days 1 and 2, the dose required to obtain the remaining AUC on day 3 of treatment could be calculated [12].

### Dose optimisation

Each patient recruited to the study received real-time dose intervention during their treatment to achieve the defined target cumulative AUC, as previously described for a comparable patient cohort [10]. In order to assess current dosing practices and to determine the most appropriate dosing regimen for infants not undergoing TDM, a retrospective analysis was performed. Predicted cumulative exposures that would have been obtained in the absence of day 3 dose adjustment, were calculated from day 1 AUC and CL values. In addition, the daily dose (mg/kg/day) required to achieve the target carboplatin AUC, was calculated for each patient. Linear regression analysis was conducted to assess the relationship between CL and body weight (BW) or age. Finally, optimised daily doses and day 1 CL data were used to predict the variability in exposure at this dose level.

Results were reported for individual patients as percent of the target AUC that they achieved. It was desirable for patients to achieve 90–110% of their target AUC, but it was considered as acceptable if patients were with 25% percent of their target AUC. Exposures achieved outside these limits were considered unsatisfactory and translate to low exposures (<75% of target AUC) and high exposures (>125% of target AUC).

### Statistical analysis

Appropriate statistical analyses were performed in order to determine any significant differences in patient exposure and dosing. A Mann-Whitney test was used to assess differences in CL and dose required to achieve target AUC in patients <5 kg and 5–10 kg. A two-way ANOVA was used to assess the impact of dose changes in patients <5 kg and 5–10 kg for a target AUC of 5.2 mg/ml.min. A Wilcoxon test was performed to assess the impact of dose changes in patients targeting an AUC of 7.8 mg/ml.min.

## Results

### Patient characteristics

Data from 82 patients (≤10 kg) were used in this analysis (1–69 weeks of age on the first cycle of TDM), the characteristics of these patients are described in Table 2. Several patients were monitored on multiple cycles of carboplatin therapy, therefore data were available for a total of 165 doses. Table 2 shows the patient characteristics from the first cycle of TDM, as patient age and weight change over multiple cycles of chemotherapy. The majority of these patients (55%) were under 3 months of age on their first cycle of TDM. The median weight of these patients was 5.13 kg and ranged from 1.6 to 10 kg. Of the 82 patients studied, 63 targeted an AUC of 5.2 mg/ml.min and 10 targeted an AUC of 7.8 mg/ml.min. The remaining 9 patients started treatment at a target AUC of 5.2 mg/ml.min, but as this exposure was well tolerated, the target was increased to 7.8 mg/ml.min with a view to improving clinical outcome. Carboplatin was used to treat a variety of tumour types, however the most common tumour types in this patient population were neuroblastoma (46%) and retinoblastoma (41%).

**Table 2:** Patient characteristics from 1st cycle of carboplatin TDM.

Characteristic	No.
Evaluable patients	82
Sex	
Male	42
Female	40
Age (weeks)	
0–4	17
5–8	17
9–12	11
13–24	12
25–52	19
52+	6
Body weight (kg)	
Median (range)	5.13 (1.6–10)
<5Kg	38
5–10Kg	44
Target AUC (mg/ml.min)	
5.2	63
7.8	10
5.2 then 7.8	9
Tumour type	
Brain tumour	1
Choroid plexus tumour	1
Clear cell sarcoma	1
Congenital glioblastoma	1
Ganglioneuroblastoma	1
High grade glioma	1
Infant medulloblastoma	1
Neuroblastoma	38
Optic pathway glioma	1
Pilocytic astrocytoma	2
Retinoblastoma	34

### Carboplatin dosing in patients < 5 kg

**Fig. 1:**
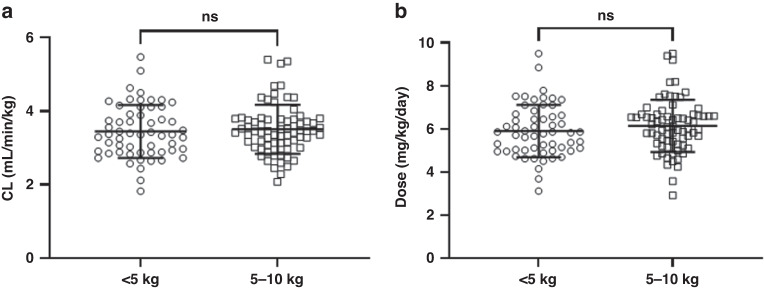
Assessing the impact of weight-based cut-offs for carboplatin dosing. Differences in (**a**) carboplatin clearance and (**b**) calculated carboplatin daily dose to achieve an AUC of 5.2 mg/ml.min between patients <5 kg and 5–10 kg. Error bars represent standard deviations.

Although there was a positive correlation between age and body weight (R^2^ = 0.56), with the group of patients <5 kg significantly younger than those 5–10 kg (*P* < 0.0001; Supplementary Fig. 1A, B), there was substantial overlap in the ages of patients between the two groups. For patients targeting an AUC of 5.2 mg/ml.min, there were no significant differences in carboplatin CL between patients <5 kg and 5–10 kg when normalised for body weight (Fig. 1a). Subsequently, there were no significant differences in the daily dose that was calculated prospectively to achieve this target AUC (Fig. 1a). Average daily doses of 5.9 mg/kg/day and 6.1 mg/kg/day were calculated for patients <5 kg and 5–10 kg, respectively, in order to achieve a target AUC of 5.2 mg/ml.min. In addition, the variability in daily dose was comparable between these two groups, with a range of 2.9–9.5 mg/kg/day required. As there was no significant difference in CL observed between these weight groups, a single recommended dose was used for the remainder of the analysis.

### Optimisation of carboplatin daily doses

**Fig. 2:**
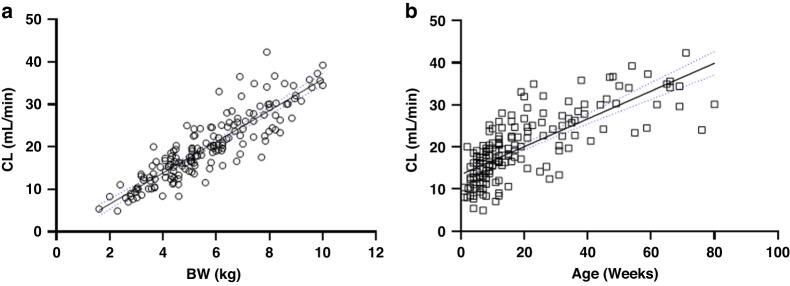
Using linear regression analysis to optimise daily carboplatin dosing. The relationship between (**a**) body weight (BW) and carboplatin CL (ml/min) (R^2^ = 0.7825) and (**b**) age (weeks) and clearance (ml/min) (R^2^ 0.5688) in 82 patients <10 kg on 165 occasions of carboplatin chemotherapy. Blue dashed lines represents the 95% confidence interval.

Linear regression analysis showed a stronger positive correlation (R^2^ = 0.78) between CL and BW (Fig. 2a) than CL and age (R^2^ = 0.57) (Fig. 2b). As BW was the more successful predictor of clearance, this was used to estimate required daily doses. Using this linear relationship, patients would require on average a dose of 1.15 mg/kg/day for every 1 mg/ml.min of targeted AUC. This equates to doses of 6 mg/kg/day and 9 mg/kg/day that would be required to target AUC values of 5.2 and 7.8 mg/ml.min, respectively, for all patients ≤10 kg.

### Comparison of optimised dose and current dosing regimens

**Fig. 3:**
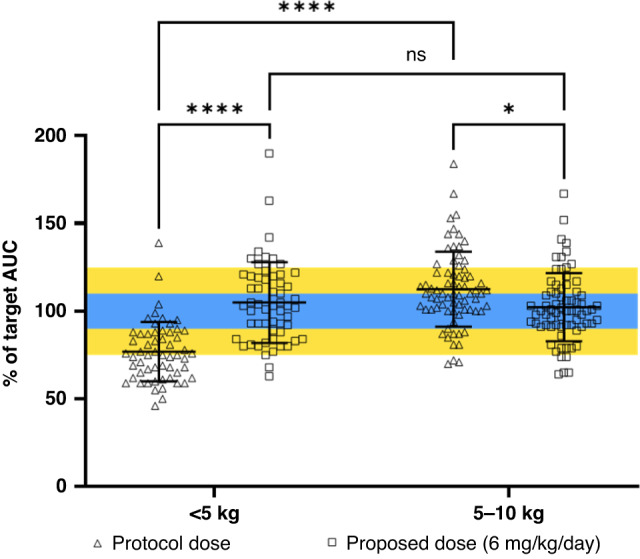
Percent of carboplatin target AUC (5.2 mg/ml.min) achieved in patients without TDM invention receiving a protocol dose of 4.4 mg/kg/day (<5 kg) or 6.6 mg/kg/day (5–10 kg) and the predicted target AUC achieved in the same patients following the proposed dose of 6 mg/kg/day. Blue shaded area represents the 10% boundaries of the target AUC achieved (desirable) and the yellow shaded area represents the 25% boundaries of the target AUC achieved (acceptable). Error bars represent standard deviation (*p < 0.5, ****p < 0.0001).

Under current dosing regimens, patients <5 kg receiving a 4.4 mg/kg/day dose achieved significantly lower carboplatin exposures than patients 5–10 kg, who were dosed at 6.6 mg/kg/day (*p* < 0.0001). However, by utilising a proposed 6 mg/kg/day dose for all patients, a significant increase in exposure (*p* < 0.0001) would be observed for those previously dosed at 4.4 mg/kg/day, as shown in Fig. 3. In contrast, a small but significant decrease in exposure (*p* < 0.05) would be observed by decreasing daily dosing from 6.6 mg/kg/day to 6 mg/kg/day for patients weighing 5–10 kg. Importantly, the proposed dosing of 6 mg/kg/day would result in no significant difference in exposure between patients <5 kg and 5–10 kg (Fig. 3).

For patients <5 kg receiving a dose of 4.4 mg/kg/day, 31/57 (54%) were within 25% of the target AUC and only 8/57 (14%) were within 10% of the target AUC (Table 3). However, by increasing the dose in these patients to 6 mg/kg/day, a greater proportion of 46/57 (81%) patients would achieve drug exposures within 25% of the target AUC. This is reflected by the calculated average exposures for these patients. At a dose level of 4.4 mg/kg/day an average of 77% of the target AUC was observed, which would increase to 105% with a proposed dose of 6 mg/kg/day. For patients 5–10 kg the average AUC achieved at 6.6 mg/kg/day was 113% of the target, above the 10% desirable limits. However, the proposed dose reduction from 6.6 mg/kg/day to 6 mg/kg/day would result in an average exposure of 102% of the target AUC in this patient group. In addition, this proposed dose change would also reduce the observed range in drug exposures observed, with 82% of patients within 25% of the target AUC, as compared to 72% at a dose of 6.6 mg/kg/day, and >50% of patients (40/72) attaining exposures within 10% of the target AUC.

**Table 3:** Number of patients within target AUC for current daily dosing regimens and proposed daily dosing regimens.

Target AUC (mg/ml.min)	Protocol Dose (mg/kg/day)	Within 10% of target AUC (% patients)	Within 25% of target AUC (% patients)	Proposed Dose (mg/kg/day)	Within 10% of target AUC (% patients)	Within 25% of target AUC (% patients)
5.2	4.4	8/57 (14)	31/57 (54)	6	20/57 (35)	46/57 (81)
	6.6	28/72 (39)	53/72 (74)	6	40/72 (56)	59/72 (82)
7.8	6.6	1/36 (3)	10/36 (28)	9	20/36 (56)	34/36 (94)
	200 mg/m^2^/day	14/36 (39)	21/36 (58)	–	–	–

**Fig. 4:**
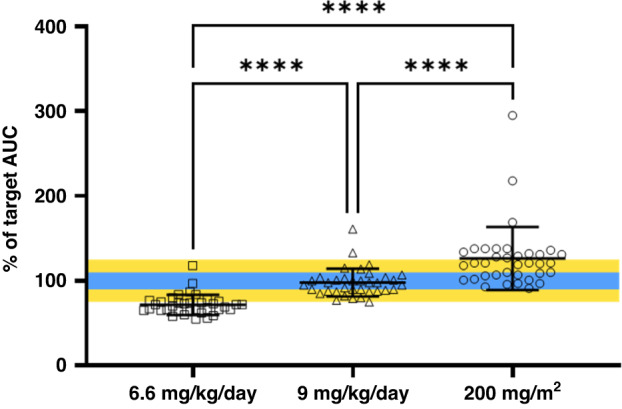
Percent of carboplatin target AUC (7.8 mg/ml.min) achieved in patients without TDM invention receiving protocol dose of 6.6 mg/kg/day and the predicted target AUC achieved in the same patients following proposed dose of 9 mg/kg/day and 200 mg/m2/day. Error bars represent standard deviation (****p < 0.0001).

Increasing daily dosing from 6.6 mg/kg/day to 9 mg/kg/day for patients targeting an AUC of 7.8 mg/ml.min, would result in a significant increase in carboplatin exposure (*p* < 0.0001) as shown in Fig. 4. At a dose level of 6.6 mg/kg/day patients achieved an average of 72% of the target exposure, below the acceptable target range of 75–125%. At this dose only one patient was within 10% of the target AUC and only 28% of patients studied were within 25% of the target AUC (Table 3). However, by increasing the dose to 9 mg/kg/day, patients would achieve an average of 98% of the target AUC (Fig. 4), with 94% of patients within 25% of the target AUC, and >50% of the patients within 10% (Table 3). In comparison, using BSA-based dosing (200 mg/m^2^/day) for this target AUC results in significantly higher exposures than observed at a dose of 9 mg/kg/day. Only 14/36 patients were within 10% of the cumulative target using this daily dosing (Table 3). In addition, 42% of patients would have exceeded the 25% target limit, resulting in excessive carboplatin exposure (Fig. 4).

## Discussion

Due to marked changes in carboplatin clearance that are commonly seen in neonates and infants in the first weeks and months of life, TDM remains the gold standard for dosing carboplatin in this patient population [10]. However, many countries do not utilise TDM treatment approaches in a childhood cancer setting, and there are few national carboplatin monitoring programmes outside of the UK. Bearing in mind the potential for an increased incidence of toxicity being associated with carboplatin use in infant patients, it is essential that dosing regimens utilised at an international level are informed by a sound pharmacological rationale.

Based on the interrogation of pharmacological data from 82 infant patients, studied across 165 cycles of treatment, we propose a body weight-based dosing approach of 1.15 mg/kg/day for every 1 mg/ml.min of targeted AUC, for all patients ≤10 kg. Where carboplatin is dosed consecutively over three days, this equates to doses of 6 mg/kg/day and 9 mg/kg/day to achieve target cumulative AUC values of 5.2 and 7.8 mg/ml.min, respectively. This proposal is based on the strong correlation (R^2^ = 0.78) observed between body weight and carboplatin CL within this patient population.

In comparison with previously published dosing algorithms, the correlation observed between body weight and carboplatin CL is a better predictor of clearance than other relationships, including EDTA clearance. While strong relationships have been reported between GFR and CL in adults, this is unlikely to be applicable to the neonate and infant patient population [11, 19]. In this respect, weight-based dosing of carboplatin has been shown to more consistently achieve target drug exposures than widely-used GFR-based methods in children 1–8 years of age [13]. The relationship between GFR and CL was not assessed in this current analysis, as very few GFR estimates were provided for the patients studied, reflecting the challenges of obtaining reliable estimates of GFR in infant patient populations. This is particularly the case for neonates dosed within the first days or weeks of life. Therefore, this analysis focused on refining carboplatin body weight-based dosing, which provides a more practical dosing approach and is currently standard of care for infant cancer patients.

Concerns of conservatively under-dosing chemotherapeutics in infant patients have recently been highlighted in a perspectives article [14]. Indeed such concerns have been substantiated for vincristine, where sub-optimal drug exposures were observed in infants dosed at <0.05 mg/kg [2, 3, 20]. In addition, a recent review by Nijstad et al. [2] showed that for 29 of the most commonly used drugs used to treat infant cancer patients, there was only sufficient evidence to recommend doses in infants for 17 of these drugs. For 14/17 of these drugs, full mg/m^2^ was recommended in infants. Only one drug (cyclophosphamide) showed age related difference in pharmacokinetics where infant patients (< 6month old) would benefit from dose reductions of 20% (mg/m^2^) [21]. TDM guided dosing was recommended for carboplatin and busulfan.

In the current study we demonstrate that current approaches to dosing carboplatin in patients <5 kg lead to suboptimal drug exposures. No significant differences in CL were observed between patients <5 kg and 5–10 kg, therefore additional dose reductions in patients <5 kg are not supported by current evidence. Moving forwards, a single mg/kg dose is proposed to support carboplatin dosing in all patients ≤10 kg, as exposures of 5.2 mg/ml.min are well tolerated in patients utilising TDM to achieve this target carboplatin exposures. Implementation of a 6 mg/kg/day dose to achieve a target AUC of 5.2 mg/ml.min in all patients ≤10 kg has the potential to reduce the incidence of patients obtaining suboptimal carboplatin exposure, particularly in the smallest patients. Equally, it has the potential to reduce the risk of patients between 5 and 10 kg experiencing excessive carboplatin exposures. To improve clinical outcome in patients where exposures of 5.2 mg/ml.min are well tolerated but sub-therapeutic, 9 mg/kg/day can be used to achieve a cumulative AUC of 7.8 mg/ml.min, as 6.6 mg/kg/day is not sufficient to achieve this target exposure (Fig. 4). Although 9 mg/kg/day can be implemented across an age range to achieve a cumulative AUC of 7.8 mg/ml.min, BSA-based dosing cannot. At a dose level of 200 mg/m^2^/day, fewer patients (58%) achieved their target AUC relative to patients dosed at 9 mg/kg/day (94%), demonstrating that the latter is a more appropriate dose to use for patients <10 kg. All of the patients in this analysis received real-time TDM of carboplatin and achieved their desired target exposures with no increase in observed toxicity relative to patients without TDM intervention (data not shown, separate manuscript in preparation). Importantly, this increased daily dose by body weight is still lower than the standard daily dose of 200 mg/m^2^/day up to a patient weight of 13 kg (Supplementary Table 1). Therefore, patients are not at an increased risk of experiencing a dose reduction when protocols switch to BSA-based dosing. This table also further illustrates the limitations of using BSA-based dosing infants, as a result of difficulties in calculating BSA in small infants. In this respect, protocols that use a percent reduction of BSA dose would benefit from switching to the appropriate mg/kg daily dose as identified here.

Although real-time monitoring of carboplatin exposure remains the best practice for dosing carboplatin in infant cancer patients, the proposed body weight-based doses have the potential to increase the number of patients achieving optimal exposures of carboplatin, where TDM is not a viable option. Future work will focus on assessing the landscape of dosing recommendations for additional drugs widely used in neonates and infants, where clinical pharmacology data remain scarce.

### Supplementary information

Supplementary data

## Data Availability

The data that support the findings of this study are available on request from the corresponding author.
