# Supplementary material for: Generation of evidence-based carboplatin dosing guidelines for neonates and infants
Source: Br J Cancer. 2023 Oct 10;129(11):1773–9. doi: 10.1038/s41416-023-02456-y (PMC10667364; doi:10.1038/s41416-023-02456-y)
Supplement: Supplementary file 1 — Supplementary data [file 41416_2023_2456_MOESM1_ESM.docx]

**Supplementary data**

**Supplementary Figure 1** A) Correlation between age (weeks) and body weight (kg) (R^2^ 0.576) and B) differences in age for patients <5kg and 5-10kg (****p <0.0001) in 82 patients studied across 165 occasions of TDM.

**Supplementary Table 1** Differences in daily dose at 9mg/kg vs 200mg/m^2^ using the CCLG nomogram for conversion of body weight to body surface area. 9mg/kg daily dosing is lower until a patient reaches 13kg

| Body Weight (kg) | BSA (m^2^) | Daily dose 9mg/kg | Daily Dose 200mg/m^2^ |
| --- | --- | --- | --- |
| 2 | 0.16 | 18 | 32 |
| 2.5 | 0.19 | 22.5 | 38 |
| 3 | 0.21 | 27 | 42 |
| 3.5 | 0.24 | 31.5 | 48 |
| 4 | 0.26 | 36 | 52 |
| 4.5 | 0.28 | 40.5 | 56 |
| 5 | 0.3 | 45 | 60 |
| 5.5 | 0.32 | 49.5 | 64 |
| 6 | 0.34 | 54 | 68 |
| 6.5 | 0.36 | 58.5 | 72 |
| 7 | 0.38 | 63 | 76 |
| 7.5 | 0.4 | 67.5 | 80 |
| 8 | 0.42 | 72 | 84 |
| 8.5 | 0.44 | 76.5 | 88 |
| 9 | 0.46 | 81 | 92 |
| 9.5 | 0.47 | 85.5 | 94 |
| 10 | 0.49 | 90 | 98 |
| 11 | 0.53 | 99 | 106 |
| 12 | 0.56 | 108 | 112 |
| 13 | 0.59 | 117 | 118 |
| 14 | 0.62 | 126 | 124 |
| 15 | 0.65 | 135 | 130 |
| 16 | 0.68 | 144 | 136 |
| 17 | 0.71 | 153 | 142 |
| 18 | 0.74 | 162 | 148 |
| 19 | 0.77 | 171 | 154 |
| 20 | 0.79 | 180 | 158 |
| 21 | 0.82 | 189 | 164 |
| 22 | 0.85 | 198 | 170 |
| 23 | 0.87 | 207 | 174 |
| 24 | 0.9 | 216 | 180 |
| 25 | 0.92 | 225 | 184 |
| 26 | 0.95 | 234 | 190 |
| 27 | 0.97 | 243 | 194 |
| 28 | 1 | 252 | 200 |
